# Statistical parametric mapping of three-dimensional local activity distribution of skeletal muscle using magnetic resonance imaging (MRI)

**DOI:** 10.1038/s41598-021-84247-0

**Published:** 2021-02-26

**Authors:** Satoshi Yamaguchi, Makoto Watanabe, Yoshinori Hattori

**Affiliations:** 1 Division of Aging and Geriatric Dentistry, Tohoku University Graduate School of Dentistry, 4-1 Seiryo-machi, Aoba-ku, Sendai, Miyagi 980-8575 Japan; 2 Institute of Living and Environmental Sciences, Miyagi Gakuin Women’s University, 9-1-1 Sakura-ga-oka, Aoba-ku, Sendai, Miyagi 981-8557 Japan

**Keywords:** Skeletal muscle, Magnetic resonance imaging

## Abstract

Analysis of the internal local activity distribution in human skeletal muscles is important for managing muscle fatigue/pain and dysfunction. However, no method is established for three-dimensional (3D) statistical analysis of features of activity regions common to multiple subjects during voluntary motor tasks. We investigated the characteristics of muscle activity distribution from the data of ten healthy subjects (29 ± 1 year old, 2 women) during voluntary teeth clenching under two different occlusal conditions by applying spatial normalization and statistical parametric mapping (SPM) to analysis of muscle functional magnetic resonance imaging (mfMRI) using increase in transverse relaxation time (T2) of the skeletal muscle induced by exercise. The expansion of areas with significant T2 increase was observed in the masticatory muscles after clenching with molar loss comparing with intact dentition. The muscle activity distribution characteristics common to a group of subjects, i.e., the active region in the temporal muscle ipsilateral to the side with the molar loss and medial pterygoid muscle contralateral to the side with the molar loss, were clarified in 3D by applying spatial normalization and SPM to mfMRI analysis. This method might elucidate the functional distribution within the muscles and the localized muscular activity related to skeletal muscle disorders.

## Introduction

The rapid increase in the elderly population and health life lost due to disability are global issues^1,2^. In recent years, the loss of skeletal muscle mass and function, known as sarcopenia, has been reported as central to the deterioration of elderly people’s independence^3^. For this reason, maintenance and recovery of skeletal muscle function through rehabilitation is important for extending a healthy lifespan. Resistance exercise is effective in rehabilitating elderly people with atrophied physical functions^4,5^, but various local activities exist within the skeletal muscle, and the function of the same skeletal muscle may differ based on the location^6^. To efficiently maintain and recover muscular function, it is necessary to apply load to muscles contributing to specific movements; therefore, it is important to measure and analyze localized activity internal to muscles involved in that movement. Additionally, although chronic muscle fatigue/pain and dysfunction is indicated as an important problem causing reduced productivity^7–9^, many points in its mechanism are still unclear. Previous studies have reported that localized activity within muscles is related to muscle fatigue and muscle pain^10–12^; hence, the measurement and analysis of localized activity were considered to contribute to discovering the cause and treatment of chronic muscle pain.

As electromyography (EMG), mainly used in studies on skeletal muscle activity and functions, measures the activity potential of skeletal muscles as an indicator of muscular activity, it has excellent temporal resolution suited to measuring muscular activity changes during exercise over time. However, to derive activity from the depths of the body requires an invasive method, such as the use of wire electrodes, and exhaustive measurements inside muscles and analysis of the functional distribution using EMG are difficult. Two-dimensional (2D) muscular activity distributions have been reported using surface electrodes allocated in an array^12–14^; however, to the best of our knowledge, there are no reports of 3D activity distributions that include deep muscle. Although the 3D distribution of glucose metabolism in muscles could be evaluated by fluorodeoxyglucose-positron emission tomography (FDG-PET)^15^, whole-body radiation exposure is a concern.

Muscle functional magnetic resonance (MR) imaging (mfMRI) has been reported as a non-EMG and non-invasive method of evaluating muscular activity^16,17^. Although the term “muscle functional imaging” covers several types of sequences and contrast, e.g., transverse relaxation time (T2), perfusion and dynamic diffusion imaging, we analyzed only the exercise-induced extension of T2 in this work. Compared with EMG, it has a low temporal resolution, but it can evaluate arbitrary regions of muscular activity, including deep areas within the body. The force exerted by the muscle must be taken into account to evaluate the muscle activity. To this end, we have reported several mfMRI studies that used teeth clenching with constant bite force as a motor task by using visual feedback from an original metal-free bite force gauge capable of monitoring the bite force in real-time in strong magnetic fields^18,19^.

An increase in exercise-induced T2 is thought to occur when decreased pH, accompanying the breakdown of glycogen and production of lactic acid in muscle cells, leads to temporary changes in osmotic pressure, causing extracellular water to move into the cells^20^. Although the exercise-induced T2 increase in skeletal muscle is also caused by blood oxygenation [the blood oxygenation level-dependent (BOLD) effect], the contribution of the BOLD effect to significant increases in T2 for several minutes after intensive exercise is smaller than that of the osmotic pressure changes^21^. Despite limited information regarding its mechanism, T2 was reported to demonstrate a high correlation with glucose uptake within the muscle in both whole muscle and per-pixel analyses^22^, and by analyzing T2 shifts before and after exercise per voxel units, analysis of local activity distributions within muscles has been reported^23^. However, as there are significant individual differences in muscle form and their internal structures^24^, and the 3-dimensional activation patterns of skeletal muscle differ significantly between subjects even when performing the same motor task^15^, there was no method of analyzing trends in local activity distribution common to multiple subjects. On the contrary, in the analysis of brain MRI, morphological standardization called “spatial normalization” has been performed by nonlinear registration of brain images in many subjects with a template image of a standard brain form, and then the common regions of signal intensity change within the subject group have been detected by statistical parametric mapping (SPM)^25–27^. To our knowledge, no study has analyzed the distribution of muscle activity using spatial normalization during voluntary motor tasks under different conditions, though a previous study has analyzed damaged muscle areas in the right thigh following electrically induced isometric contractions^28,29^. By using a standard template image of the entire head and neck and by performing spatial normalization and statistical mapping on masticatory muscles that fit entirely in the head and neck MRI, it may be possible to analyze the characteristics of local activity distribution in masticatory muscles in voluntary movement tasks under different conditions.

This study aimed to statistically elucidate the characteristics of the distribution of muscle activity common to a group of subjects with voluntary movements under different conditions by applying spatial normalization and SPM to the analysis of mfMRI with teeth clenching tasks under different occlusal conditions. Then, to clarify the advantages of using our method, we analyzed the spatially normalized MRI by using two conventional methods (i.e., comparing the mean T2 of muscles and extracting activity regions for each test subject using the mean and standard deviation within muscle T2), as well as SPM.

## Methods

### Ethical approval

The aims, method, and safety of the study were fully explained to the test subjects, and their written informed consent was obtained. Approval was obtained from the Tohoku University Graduate School of Dentistry Ethics Committee (No. 24-32). The study conformed to the standards set by the Declaration of Helsinki, except for registration in a database.

### Study participants

Ten subjects with healthy teeth and jaws (mean age ± standard deviation, 29 years ± 1; two women) participated in the present study. The exclusion conditions included a functional abnormality or history of such in the stomatognathic system, conditions derived from the teeth or tissue surrounding the teeth, those with neural/muscular/metabolism-related conditions, and those contraindicated for MRI. The test subjects were the same in a separate previous study, and we reanalyzed the image data of the previous work^19^. The effect size (dz) of the T2 change of all masticatory muscles after clenching (single tooth clenching on the left first molar at 40% of the maximum voluntary clenching force for 1 min) was 0.86 in our previous study of other subjects^30^. A power analysis to determine the required sample size for one-sided test using G*Power3^31^, assuming an effect size of dz = 0.86 and an error probability of α = 0.05, showed that 10 participants were sufficient to reach a statistical power 1 − β = 0.8.

### Bite force gauge

**Figure 1:**
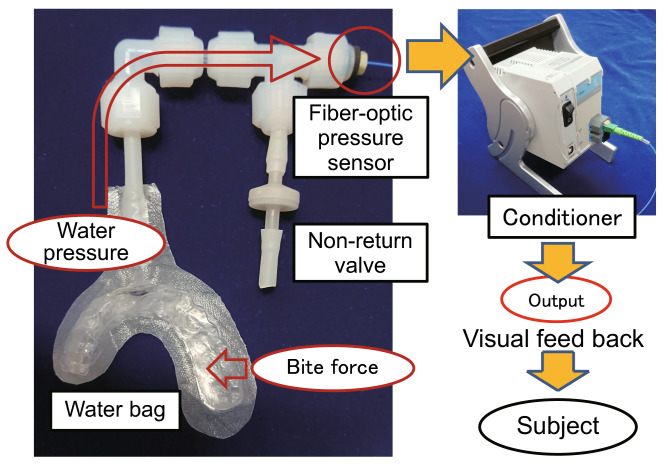
Overview of the metal-free bite force gauge (simulated left molar loss) and experimental set-up for visual feedback.

We applied fiber-optic compact pressure sensors (FOP-M-BA; FISO Technologies Inc., QC, Canada) to manufacture a bite pressure meter that could be used in strong magnetic fields (Fig. 1)^19^. A resin bag, formed to match the dental arch of each subject, was joined with plastic tubes in which pressure sensors have been embedded, and by filling them with water internally, we measured changes in water pressure based on the bite strength. There are two types of resin bags, reproducing the bite states of “intact dentition” and “simulated left molar loss”. By simultaneously measuring the external force applied by the force gauge (ZP-1000N; IMADA Co., Ltd., Toyohashi, Japan) and the internal water pressure, for each bite pressure meter created, we obtained a calibration line for calculating the bite force from water pressure^19^. No occlusal contact of the teeth in the simulated molar loss side was confirmed using occlusal examination material.

### Exercise tasks

A thermoplastic sheet (thickness: 0.5 mm) was made into a dental splint to match the maxillary dental arch of each subject, and the resin bag was fixed to the maxillary dental splint. Each subject was told to do the exercise tasks of teeth clenching for 1 min with two types of resin bags (two conditions of “intact dentition” and “simulated left molar loss”). Using the calibration line, the water pressure at 40% of maximal voluntary clenching (40% MVC) was calculated from the water pressure of 100% MVC. The subject was told to maintain the water pressure at 40% MVC during clenching by visual feedback of the force gauge.

The pressure sensor was connected, via a 25 m fiber-optic cable, to a conditioner (EVO-SD-2; Fiso Technologies Inc. QC, Canada) in the operations room, and using the laptop PC connected to the conditioner, the bite force of the subject in the gantry could be measured in real-time (Fig. 1). The measured values were displayed on a screen in the capture room using a projector and fed back through a mirror attached to a head coil to the subject^19^.

### MRI protocol

MR images were captured at rest times before and immediately after the exercise tasks. All MRI scans were performed using the Signa HDxt (1.5T) (GE Healthcare, Waukesha, WI) installed in Sendan Hospital (Tohoku Fukushi University) with an eight-channel brain coil (8HRBRAIN). The subjects were in a dorsal position, and their heads were fixed to a head coil. Bite force gauge was fixed to the head coil using medical tape. The parameters of the 2D conventional spin-echo sequence were as follows: repetition time (TR) = 2500 ms; echo time (TE) = 20/80 ms; flip angle = 90°; 22 slices, with slice thickness = 3 mm; slice gap = 0 mm; matrix = 256 × 256; field of view = 200 mm; pixel size = 0.78 × 0.78 mm; and scan time = 7 min 10 s.

### Image analysis

#### T2 calculation

All MR images were converted to Nifti format using dcm2nii (Chris Rorden: http://people.cas.sc.edu/rorden/mricron/dcm2nii.html). Image processing software Fiji was used to reconstruct T2 images^32^. When calculating T2, the following formula was applied concerning each voxel.

$$ {\text{T2}} = \left( {{\text{t}}_{{\text{b}}} - {\text{t}}_{{\text{a}}} } \right)/{\text{In}}\left( {{\text{I}}_{{\text{a}}} /{\text{I}}_{{\text{b}}} } \right), $$

(t_a_ and t_b_ indicate the spin-echo times, and I_a_ and I_b_ indicate the signal intensity for TE = 20 and 80).

As the T2 images are reconstructed by one from the TE20 and 80 images, a total of 40 T2 image data sets were obtained (10 test subjects × 2 conditions × before and after task).

#### Spatial normalization of the T2 maps

**Figure 2:**
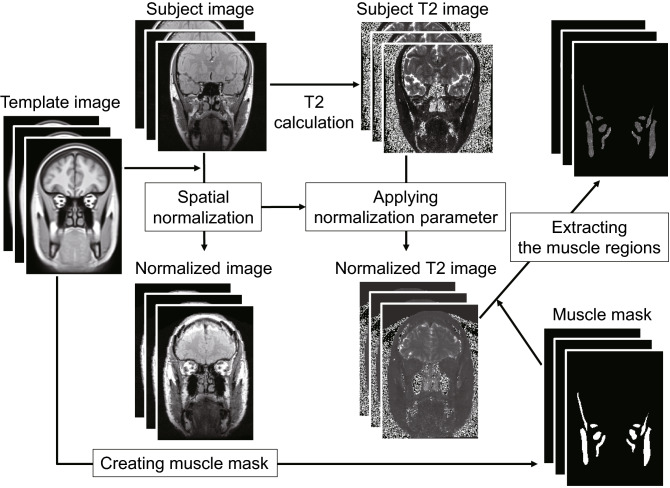
Pipeline of the spatial normalization of the T2 image to the template image and extracting the muscle regions with a mask image. The transformation parameter for performing nonlinear registration of the TE20 image to the template image was applied to the corresponding T2 images.

Using Advanced Normalization Tools (ANTs), which have a strong reputation in the 3D nonlinear registration of brain images^33^, spatial normalization was performed on the whole head and neck section (Fig. 2). For template images used as reference, images containing the whole head and neck section were selected (T_template2.nii.gz in IXI.zip) from the many brain MR image templates (https://figshare.com/articles/ANTs_ANTsR_Brain_Templates/915436)^34^.

T2 images reflecting muscular activity have a large amount of noise and low contrast, so it is difficult to perform nonlinear registration directly. Therefore, we first used TE20 images, which have low noise, strong contrast, and rich anatomical information, and the transformation parameter for performing nonlinear registration to the head and neck section template image was calculated for all TE20 images. By applying this transformation parameter to the corresponding T2 images, spatial normalization of the T2 image to the template was performed. The above processing was executed using antsRegistration and antsApplyTransforms contained in the ANTs package.

##### T2 image segmentation and removing outliers

By tracing the masticatory muscle outline of the head and neck template image used in spatial normalization, we manufactured a masticatory muscle mask image. All muscle outlines were drawn by a dentist (S.Y.) who has been engaged in masticatory muscle MRI studies for 15 years. By applying this mask image to the T2 image after spatial normalization, we were able to extract only the masticatory muscle region. The background value used in the masked regions was 0. According to the previous review, the muscle tissue T2 is reported to be approximately 27–44 ms, and the fat tissue T2 is generally > 100 ms^35^. Considering the extent of muscle tissue T2 after exercise, values of the voxels with a value of 100 or above were converted to 0. By doing this and by applying Implicit Mask, we excluded voxels with inappropriate values for muscle tissue included in the T2 images from spatial smoothing in the SPM analysis. To evaluate the effect of this exclusion, the percentage of excluded voxels from the masticatory muscle volumes of interest (VOIs) was calculated. For this processing, we used ImageMath, included in the ANTs package.

#### Dice similarity coefficient calculation

We calculated the dice similarity coefficient (DSC) using the following formula to verify the accuracy of 3D nonlinear registration to the head and neck template images^36^.

$$ {\text{DSC}}\left( {{\text{A}},{\text{B}}} \right) \, = {\text{ 2N}}\left( {{\text{A}} \cap {\text{B}}} \right) \, / \, \left[ {{\text{N}}\left( {\text{A}} \right) \, + {\text{ N}}\left( {\text{B}} \right)} \right]. $$

A expresses the mask region of the image which is transformed from the mask image created by manually tracing the masticatory muscle form in the TE20 image by the transformation parameter for spatial normalization, while B expresses the mask region of the mask image created by manually tracing the masticatory muscle form in the head and neck template image. N(A) is the number of voxels included in A, N(B) is the number of voxels included in B, and N(A ∩ B) is the number of voxels common to A and B. The manufacturing of mask images, image calculation to detect common areas, and voxel number count was executed using Fiji. DSC(A,B) was calculated for all 40 images, and statistical analysis was performed using the Friedman test on whether there was a significant difference among the total of four conditions (before and after clenching under two conditions of “intact dentition” and “simulated left molar loss”).

#### Calculation of mean T2 and standard deviation within muscles

By applying the VOI created from the various masticatory muscle forms in the template to all T2 images after spatial normalization and segmentation, we calculated the mean T2 and standard deviation within the VOI of each masticatory muscle for all 40 T2 images.

#### Mean T2 statistical analysis

Using SPSS statistics 22.0 (IBM Japan, Tokyo, Japan), we analyzed the increase of mean T2 within the masticatory muscle VOI induced by teeth clenching. As normality of the mean T2 was not recognized for some VOI, we used a non-parametric method for all statistical analyses. We tested whether there was a significant increase within all VOI using the Wilcoxon signed-rank test. The level of significance for the statistical processing was set to 5%.

#### Muscular activity distribution analysis for each subject

Based on the conventional method reported by Kinugasa et al.^23^, the distribution of masticatory muscle activity for each subject was analyzed for T2 images after spatial normalization. The activity regions (voxels) were defined by the ranges of voxels with T2 values greater than the mean T2 + 1SD of the relevant VOI in each rest image and lower than the mean T2 + 1SD of the relevant VOI in each task image, in spatially normalized images after the task. Additionally, an indicator of the activity in these activity regions was obtained by subtracting the mean T2 of the masticatory muscles, including these voxels at rest condition from the value of voxels in the activity regions. Finally, we applied a 3-mm FWHM Gaussian filter to improve the visibility of the distributed image and create a fusion image with the template image.

#### SPM analysis

**Figure 3:**
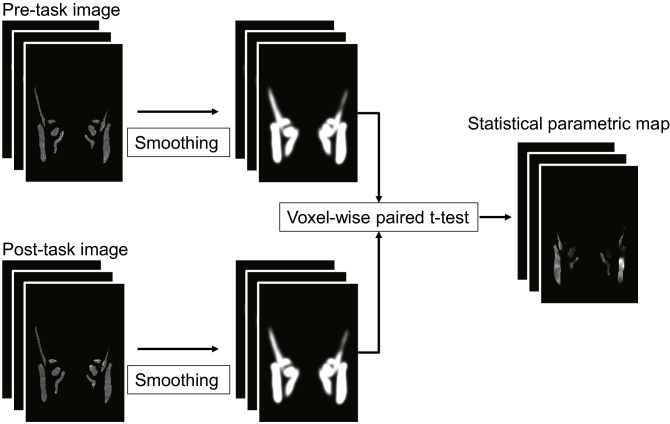
Voxel-wise statistical analysis of spatial normalized T2 images. Smoothing was applied using a 6-mm FWHM Gaussian filter.

We used SPM8 (Wellcome Institute, London, UK) to create statistical parametric maps of T2 changes within the masticatory muscles after an exercise task (Fig. 3). First, we applied a 6-mm (double of slice thickness) FWHM Gaussian filter to T2 images after spatial normalization, segmentation (the background value was 0), and removing outliers (converting outliers to 0), with “Implicit Mask” which excluded voxels below 0 from the analysis. The voxel-wise comparison of the T2 was performed by a paired t-test between before and after the clenching task. A mask image created by manually tracing the masticatory muscle of the template image was used as the “Explicit Mask.” The initial voxel threshold was set to 0.001 uncorrected. Clusters were considered as significant when falling below a cluster-corrected p(FWE) = 0.05. Statistical parametric maps of T2 represented the region of significant-high activity within the muscle common to the group of subjects. Finally, voxel-wise maps of the effect size *d* were created from the output of the T-value image by SPM analysis^37^. When calculating d, the following formula was applied in relation to each voxel^38^, where t represents the voxel values of the T-value images, and n represents the number of subjects.

$$ {\text{Cohen`s}}\;d =  t/{\sqrt {\text{n}} }. $$

Effect size maps showed the intensity of the activity in various regions within the muscle of the group of subjects.

## Results

### Spatial normalization of the T2 map

**Figure 4:**
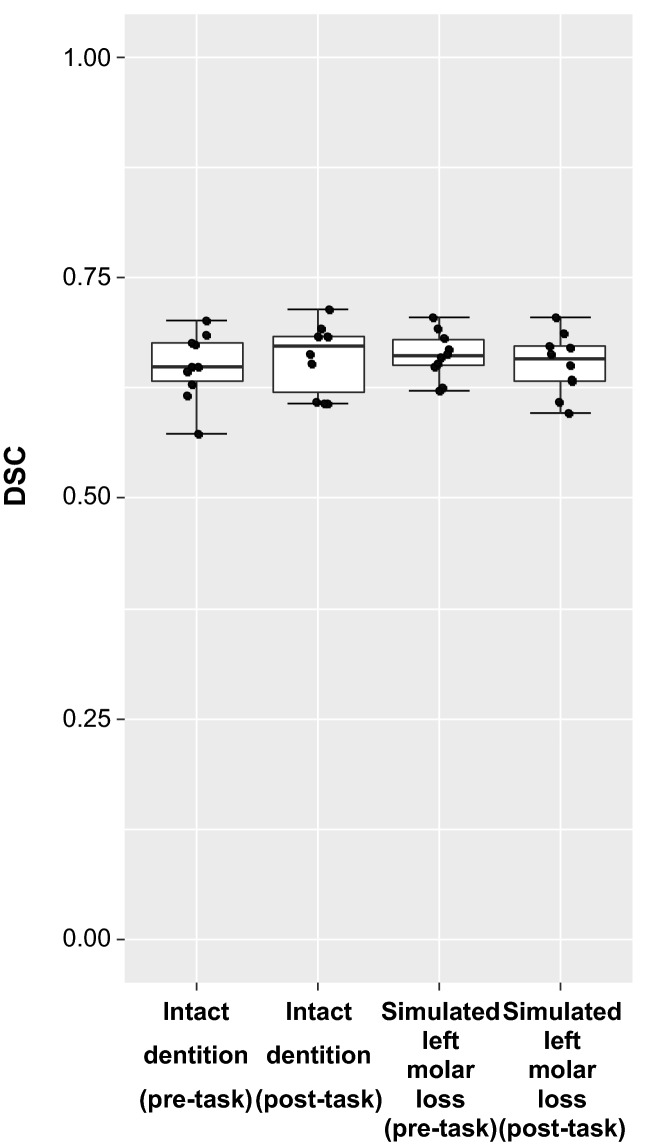
Box-and-whisker diagram of the dice similarity coefficient (DSC). There was no statistical difference among the four conditions.

Figure 4 shows a box-and-whisker diagram of DSC. On the Friedman test, no significant difference could be observed in DSC under the four conditions (p = 0.840). The median values were 0.65 and 0.67 before and after the “intact dentition” clenching exercise, respectively, and it was 0.66 for both before and after the “simulated left molar loss” clenching.

### Changes in mean T2 within each masticatory muscle

**Figure 5:**
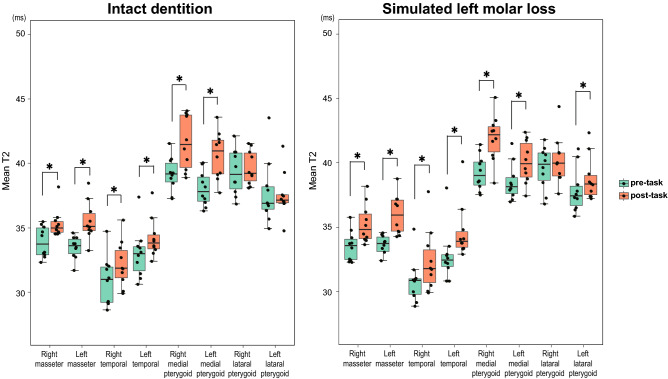
Comparison of the pre- and post-clenching mean T2 (ms) calculated from each VOI. *p < 0.05 (Wilcoxon signed-rank test). Significant increases in mean T2 were detected in all muscles between the pre- and post-tasks except for the lateral pterygoid muscles in the “intact dentition” clenching and the right lateral pterygoid muscles in the “simulated left molar loss” clenching.

Figure 5 shows the mean T2 box-and-whisker diagram within each masticatory muscle. On the Wilcoxon signed-rank test, significant increases of mean T2 were detected in all muscles between pre-task and post-task except for the lateral pterygoid muscles in the “intact dentition” clenching and the right lateral pterygoid muscles in the “simulated left molar loss” clenching.

### Distribution of T2 extension for each test subject

**Figure 6:**
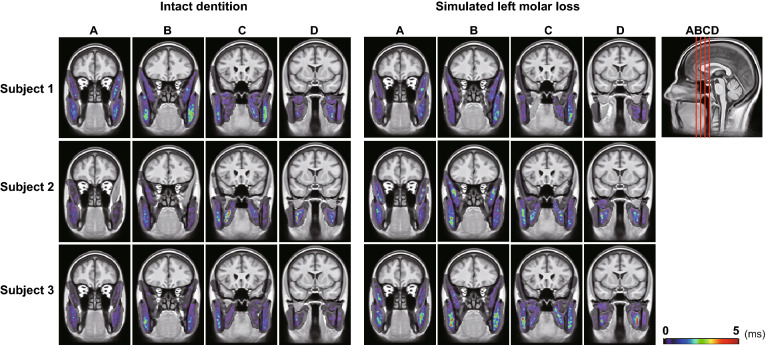
Muscle activity distribution images of three representative subjects. Four slices are shown at 10 slice intervals. There is a large distribution difference between subjects, and no specific tendency is observed.

The resulting images of the three representative subjects are shown in Fig. 6. Despite having a similar task, the activity distribution of the masticatory muscles is widely varied.

### Percentage of voxels excluded from SPM analysis

The mean percentages in the masticatory muscle VOIs of voxels excluded with Implicit Mask from the SPM analysis were 0.63 ± 0.49% and 0.56 ± 0.27% before and after the “intact dentition” clenching exercise, respectively, and those before and after the “simulated left molar loss” clenching were 0.43 ± 0.26% and 0.71 ± 0.45%.

### T2 statistical map of the masticatory muscles created from all subject’s images

**Figure 7:**
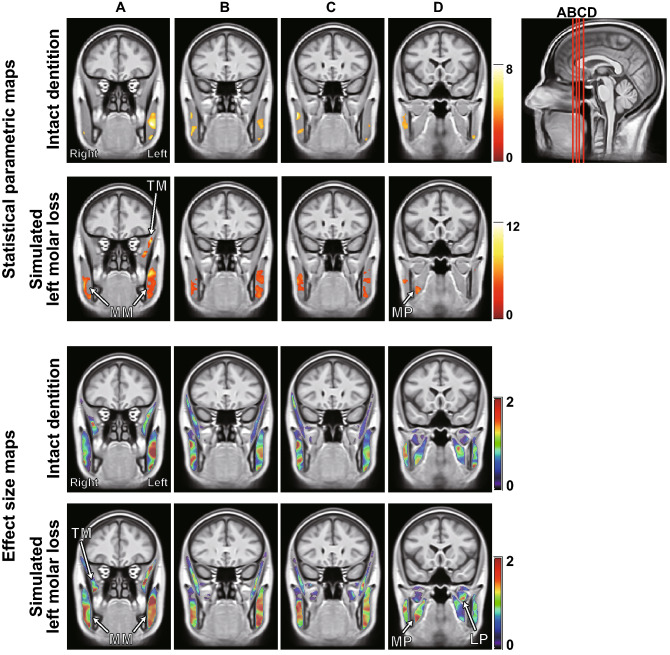
Statistical parametric maps [cluster-corrected p (FWE) < 0.05] and effect size (Cohen’s d) maps of T2 in all masticatory muscles. Four characteristic slices are shown at different slice gaps. The color bar represents the t-value or effect size (Cohen’s d). The area of significantly increased T2 was larger after the “simulated left molar loss” clenching than after the “intact dentition” clenching in the statistical parametric maps. The masseter muscle of the occlusal support defect side (left) was characterized by red and yellow areas that spread throughout the muscle in the effect size (Cohen’s d) maps. *LP* lateral pterygoid muscles, *MM* masseter muscles, *MP* medial pterygoid muscles, *TM* temporal muscles.

T2 statistical maps (FWE cluster-corrected, p < 0.05) and effect size (Cohen’s *d*) maps superimposed on the template are shown in Fig. 7, and the statistics per cluster are shown in Table 1. In the “intact dentition” clenching, areas with significant T2 increase were small (total cluster size: 5785 voxels) and located near the center of the masseter muscle. By contrast, in the “simulated left molar loss” clenching, areas with significant T2 increase enlarged (total cluster size: 13,161 voxels) and were observed in the occlusal support side of the medial pterygoid, the inferior part of the masseter muscles, and in the occlusal support defect side of the temporal and deep masseter muscles. On the effect size maps, red and yellow areas extended throughout the masticatory muscles of the occlusal support defect side, and a high effect size area was also observed in the lateral pterygoid muscle.

**Table 1:** All significant clusters of T2 in all masticatory muscles.

Condition	Muscle		Cluster			
			p (FWE-corrected)	Size (voxels)		
Intact dentition	Right	Masseter muscle (upper)	0.009	915	1497	5785
		Masseter muscle (lower)	0.033	582		
	Left	Masseter muscle (upper)	< 0.001	3651	4288	
		Masseter muscle (lower)	0.026	637		
Simulated left molar loss	Right	Masseter muscle	< 0.001	3428	3971	13,161
		Medial pterygoid muscle	0.029	543		
	Left	Masseter muscle	< 0.001	8219	9190	
		Temporal muscle	0.004	971		

## Discussion

To the best of our knowledge, this is the first report that analyzed voluntary motor tasks under different conditions for the local activity distribution in the skeletal muscle that is common in the subject group, by applying spatial normalization with a template image and statistical parametric mapping. In our previous study, the contribution of each muscle to the exertion of occlusal force was clarified by analyzing the correlation between the total T2 change of the entire VOI set for each masticatory muscle and occlusal force, but the distribution of activity within the VOI was unknown^19^. In this study, although we used almost the same MR data, we applied spatial normalization and SPM to comprehensively analyze T2 changes inside the muscle in voxel units, thereby revealing the characteristics of the activity distribution common to the subject group rather than the amount of activity. The activity distribution in the masticatory muscles differed greatly depending on the slight variation in the presence or absence of occlusal support of the left molar part.

Masticatory muscles are important for stomatognathic function and have been the subject of many dental studies conducted mainly by surface EMG. Masticatory muscles are fractionated by aponeurosis, and their internal structures vary greatly depending on the person^24^, and different local activity distributions are thought to exist within the muscles depending on the subject. Against this background, some recent studies have reported that the local activity of the masseter muscle was measured two-dimensionally using array electrodes^13^. Testa et al. analyzed the local activity distribution in the masseter muscle during tooth clenching with maximum occlusal force using array electrodes arranged in 13 rows and 5 columns at 8-mm intervals, and such distribution was compared between patients with chronic neck pain and healthy subjects^12^. Patients with chronic neck pain were reported to show higher masseter activity levels, and different distributions of activities than healthy subjects, and the position of the center of activity was located more cranial and more anterior in the patient group. However, the 3D local activity distribution was unknown, and even for the 2D distribution, only visual qualitative evaluation of the representative subject's activity map was performed, except for the position analysis of the center of activity. Qualitative assessments alone could not define the normal state of the local activity distribution in the muscle and compare quantitatively or statistically with patients. 2D measurement of local muscle activity using a similar array electrode for patients with shoulder myalgia has also been reported^10^, and the relationship between local muscle activity and dysfunction is important in understanding the whole-body skeletal muscle. The analysis method presented herein might help add new knowledge to this area.

In this study, spatial normalization must be performed with stable accuracy. No statistically significant difference in the DSC of the four conditions was found (Fig. 4), and the median value of all four conditions ranges from 0.6 to 0.7. Although the DSCs in the present study were low compared to recent brain imaging studies, these were not low compared with previous reports on spatial normalization accuracy, especially in the hippocampus and basal ganglia region on early brain functional imaging^39–41^ and registration accuracy with medical images of various organs^42,43^. In many cases, the masticatory muscle of the subject image protruded from these outlines of the template at the pterygoid muscles or the posterior part of the masseter muscles, and some voxels might be excluded from the analysis at these sites. In the future, analysis accuracy may improve due to the development of the spatial normalization method. However, the percentages of voxels in the VOIs of masticatory muscles that were excluded with Implicit Mask from the SPM analysis were approximately 0.6% in all conditions. This is considered to have a limited impact on the results.

In the comparison of the intramuscular mean T2, a significant increase was not detected in the lateral pterygoid muscles in the “intact dentition” clenching and the occlusal support side lateral pterygoid muscle in the “simulated left molar loss” clenching (Fig. 5). The lateral pterygoid muscle that pulls the mandibular condyle forward and stabilizes the mandible might be barely stimulated^44^ because the mandibular condyle did not move upward when the occlusal support was present in the molar^45^. In other muscles, the mean T2 was significantly increased in both conditions, but differences due to task conditions were unclear. A previous study that performed VOI analysis without spatial normalization on the same data set showed a significant T2 increase in all masticatory muscles except for the superior head of the right lateral pterygoid muscle after the “intact dentition” clenching^19^. In the analysis of previous studies, the lowest value from all clenching conditions was used as the mean T2 value at rest, so it is not possible to make a simple comparison with the results of this study, which compared the mean T2 values before and after each exercise. However, even when VOI analysis was performed on the T2 images after spatial normalization using a common VOI set drawn on the template image, a significant increase in the mean T2 could be detected in most muscles except for the lateral pterygoid muscle, as was the case in the previous study in which analysis was performed by manually setting the VOI of the masticatory muscles for each MR image. The lateral pterygoid muscle might be relatively small and have high difficulty in spatial normalization. Although it was likely that some voxels were excluded from the current analysis due to low DSC, the results of the VOI analysis in this study might well reflect the T2 changes in the whole muscle. It is significant that, in this study, the analysis was automated, eliminating the time cost and bias of manually setting the VOI many times. As a result of the distribution analysis of the T2 extension for each subject (Fig. 6), the local activity distribution within the masticatory muscles varies greatly among individuals, and it seems difficult to find common features from the qualitative comparison.

In the statistical T2 map (Fig. 7) and table of cluster size (Table 1), the area of significantly increased T2 was larger after the “simulated left molar loss” clenching than after the “intact dentition” clenching. We considered that common areas in the muscle of many subjects were activated to stabilize the mandible with an unstable occlusion during the “simulated left molar loss” clenching. In particular, the mandibular condyle of the defect side was displaced upward during clenching with a unilateral occlusal support defect in the previous report^45^. In this study, a significant increase in T2 was not detected with correction for multiple comparisons at the cluster level, probably because the lateral pterygoid muscle is smaller than the other masticatory muscles. However, the lateral pterygoid muscle on the simulated molar defect side showed a high effect size. The activity of the lateral pterygoid muscle of the defect side might contribute to the stabilization of the mandible by pulling the condyle forward. Our previous mfMRI study showed a significant increase in the activity of the ipsilateral lateral pterygoid muscle by the forward displacement of the occlusal contact point during unilateral tooth clenching^30^. In this study, a local activity observed only in the lower part of the medial pterygoid muscle on the occlusal support side is a finding that supports the internal heterogeneous local activity confirmed by the previous EMG study^46^. In addition, the masseter on the occlusal support side was divided into two active areas vertically, which is consistent with the results of the 2D activity distribution of the masseter measured by array electrodes in a previous study^12^. In the masseter muscle of the defect side, the large area of significant increase on the parametric map and red and yellow areas extending throughout the muscle on the effect size map suggests that the molar occlusion support defect may expand the active area inside the ipsilateral masseter muscle. This 3D expansion of the common active area is a new finding that could not be detected by conventional methods and suggests that loss of occlusal support in the molars may cause muscle fatigue and pain in the ipsilateral masseter muscle. This might support previous reports that posterior tooth loss is associated with temporomandibular disorders, with masticatory muscle pain as one of the primary symptoms^47^. Statistical analysis of the 3D distribution of the muscle activity inside the masticatory muscle has not been reported in previous studies, and it is completely new information, so there is a limit to verification. However, the muscles in which the significant active areas were detected are considered appropriate compared with those in previous reports.

Although VOI analysis allows quantitative comparison of activity across the entire VOI, strong localized activity may be overlooked because T2 changes within the VOI (often the entirety of the anatomically fractionable muscle) are averaged. While local activity assessment is possible by using the divided VOI, the manual division is a deliberate manipulation and does not necessarily reflect the natural distribution of activity. Since SPM comprehensively analyzes local T2 in voxel units across multiple muscles of interest, it is possible to extract local activity larger than voxel size without bias and to elucidate its distribution. Referring to the present results, the comparison of the mean T2 within VOI did not reveal clear features between conditions and between left and right in the medial pterygoid muscle (Fig. 5), but the SPM and effect size map results clearly detected local activity with different distribution between left and right (Fig. 7).

There is a high possibility that our method can be applied to the analysis of skeletal muscle activity other than masticatory muscles, and it is considered that the statistical analysis of the activity regions within the muscles common to all test subjects by condition can contribute to elucidating the functional distribution within the muscles and intramuscular local activities related to skeletal muscle disorders. In the future, if we can establish a method for analyzing muscle activity by applying spatial normalization and collect a large amount of data to create a 3D database of muscle activity distributions for healthy subjects, it is expected to be a new clinical examination that can objectively evaluate changes in muscle metabolism and abnormal muscle activity associated with muscle dysfunction or aging. To achieve this, it is necessary to study the effects of fat infiltration and other factors by calculating a more accurate T2 using a multi-TE spin echo^48^. Meanwhile, for 3D analysis of intramuscular activity distribution, it is necessary to obtain high-resolution MR images with a large number of slices, which makes imaging with more than three multi-TEs difficult. The two TEs used in this study were determined based on previous studies^23^, but more appropriate TEs need to be examined to reduce noise in T2 images.

This study has some limitations. First, masticatory muscle activity may have differed from the subjects’ clinical masticatory muscle activity during clenching because of the increase in occlusal height diameter caused by wearing the bite force gauge. Second, there were only 10 subjects. Although the number of subjects required to compare the intramuscular mean T2 calculated by the power analysis is satisfied, more subjects are required to perform the voxel-wise analysis by SPM accurately. Third, although spatial smoothing using a Gaussian filter is common in functional neuroimaging, it cannot be ruled out that the introduction of spatial correlation may have induced false positives in analyses that infer assumptions of independence in voxel observations. In the future, it is necessary to increase the number of subjects to improve the reliability of results.

In conclusion, the characteristics of the muscle activity distribution common to a group of subjects during clenching, especially the active region in the temporal muscle ipsilateral to the side with the molar loss and the medial pterygoid muscle contralateral to the side with the molar loss, and the expansion of the active region in the masseter muscle ipsilateral to the side with molar loss could be clarified in 3D by applying spatial normalization and SPM to mfMRI analysis. This method may enable us to statistically obtain information about the functional localization inside the muscle that could not be obtained by EMG or traditional mfMRI analysis. If a database of normal muscle activity distribution can be created, it may contribute to the detection of abnormal muscle activity associated with various muscle dysfunctions.

## Data Availability

MRI datasets generated during and/or analyzed during the current study are available from the corresponding author on reasonable request.

## Code availability

Scripts used for data analysis are available upon reasonable request.
